# Exploring causal components of plasticity in grey seal birthdates: Effects of intrinsic traits, demography, and climate

**DOI:** 10.1002/ece3.6787

**Published:** 2020-09-28

**Authors:** William Don Bowen, Cornelia E. den Heyer, Shelley L. C. Lang, Damian Lidgard, Sara J. Iverson

**Affiliations:** ^1^ Population Ecology Division Bedford Institute of Oceanography Dartmouth NS Canada; ^2^ Department of Biology Dalhousie University Halifax NS Canada

**Keywords:** AMO, birthdate, breeding phenology, grey seal, NAO, offspring weaning mass, plasticity

## Abstract

Change in breeding phenology is often a response to environmental forcing, but less is known of the mechanism underlying such changes and their fitness consequences. Here, we report on changes in the breeding phenology from a 27‐year longitudinal study (1991–2017) of individually marked, known‐aged grey seals (*Halichoerus grypus*) on Sable Island, Nova Scotia, Canada. We used generalized linear mixed models and a 3‐step process to develop a model that includes interactions between intrinsic and extrinsic covariates and to test hypotheses about the influence of fixed factors (maternal age, parity, previous reproductive success, pup sex, colony density, Atlantic Multidecal Oscillation (AMO), North Atlantic Oscillation (NAO), and Sea Surface Temperature) and a random factor (female identity) on parturition dates. We also examined the consequences of the shift in birthdates on maternal energy allocation in offspring as measured by pup weaning mass. Birthdates were known for 2,768 pups of 660 known‐age females. For 494 females with ≥2 parturition dates, repeatability as measured by the intraclass correlation was high (mean = 0.66). 87% of the variation in birthdates was explained by a mixed‐effects model that included intrinsic and extrinsic fixed effects. Most of the explained variation was associated with the random effect of female identity. Parity was the most important intrinsic fixed effect, with inexperienced mothers giving birth later in the season than multiparous females. Over almost 3 decades, mean birthdates advanced by 15 days. The mixed model with intrinsic effects and population size, the detrended AMO from the previous year and mean NAO in the previous 3 years explained 80% of the variation with 21% of variation from the fixed effects. Both primiparous and multiparous individuals responded to the climate forcing, and there was strong evidence for heterogeneity in the response. Nevertheless, the shift in birthdates did not impact pup weaning mass.

## Graphical abstract

Here, we studied how breeding phenology responds to maternal traits and demographic and environmental conditions over almost three decades and the resulting fitness consequences of those changes in a well‐studied population of grey seals. Mean birthdates advanced by 15 days. Longitudinal data on over 500 individual females showed that maternal birthdates were highly repeatable but despite that repeatability females responded to environmental forcing. Both primiparous and multiparous individuals responded to forcing by AMO, but there was strong evidence for heterogeneity in the response. Despite changing birthdates offspring weaning mass was not affected showing that climate driven changes in breeding phenology may not always have measurable consequences.

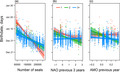

## 1 INTRODUCTION

The timing of births can have important consequences for traits affecting maternal reproductive success and survival (Charmantier et al., 2008; Cote & Festa‐Bianchet, 2001; Dunn & Winkler, 1999). Variation in breeding phenology has been found in many taxa and has generally been interpreted as a common response of individuals to environmental change (Beebee, 1995; Crick, Dudley, Glue, & Thomson, 1997; Forchhammer, Post, & Stenseth, 1998; Post & Stenseth, 1999; Thackeray et al., 2010). However, changes in breeding phenology could also be influenced by changes in population age structure and density, which could serve to either reinforce or dampen environmental drivers (Lunn, Boyd, & Croxall, 1994; van de Pol, Osmond, & Cockburn, 2012), and by the physiological state of individuals, previous reproductive history, and food availability (Boyd, 1984; Hamel, Côté, & Festa‐Bianchet, 2010; McNamara & Houston, 1996; Trillmich & Ono, 1991). Despite the number of studies on breeding phenology, we still have relatively little understanding of the mechanisms underlying such changes and their fitness consequences for individuals (Cordes & Thompson, 2013; Rotella, Paterson, & Garrott, 2016; Stopher, Bento, Clutton‐Brock, Pemberton, & Kruuk, 2014).

Changes in breeding phenology could arise through changes in the breeding population brought about through recruitment, immigration, genetic changes resulting from selection or drift, or phenotypic plasticity (Przybylo, Sheldon, & Merila, 2000). Phenotypic plasticity could be manifested by all females responding in a similar way to an environmental driver. On the other hand, newly recruiting females might give birth earlier or later than experienced females in response to the same driver, as multiparous females might be better able to buffer the effects of such changes on implantation and fetal growth (Nussey, Clutton‐Brock, Albon, Pemberton, & Kruuk, 2005; Nussey, Clutton‐brock, Elston, Albon, & Kruuk, 2005; Rotella et al., 2016). Thus, there may be intra‐specific variation in fitness in response to environmental drivers (Visser et al., 2003) along a continuum from uniform response of all females to selective response depending on the ability of a female to buffer change. As both intrinsic (individual traits) and extrinsic (environmental conditions) factors may interact to drive changes in birth phenology, studying these factors together may provide deeper insight (Coulson, Milner–Gulland, & Clutton–Brock, 2000; Rotella et al., 2016).

To date, the response of breeding phenology to environmental change has been most thoroughly studied in birds (e.g., Crick et al., 1997; Forchhammer et al., 1998; Frederiksen, Harris, Daunt, Rothery, & Wanless, 2004), amphibians (BeeBee, 1995; Forchhammer et al., 1998), and ungulates (Post & Stenseth, 1999). In most pinnipeds, the timing of birth is highly synchronized and late births can result in lower offspring weaning mass and juvenile survival (Hall, McConnell, & Barker, 2001; Boness, Bowen, & Iverson, 1995; Bowen, den Heyer, McMillan, & Iverson, 2015). Large shifts in the distribution of birthdates have been reported in several pinniped species apparently in response to improved foraging during gestation (Reijnders, Brasseur, & Meesters, 2010) and decreased population density (Cordes & Thompson, 2013).

As many studies use cross‐sectional data, less is known about how the birth or laying dates of individuals respond to environmental change. However, a growing number of longitudinal studies are providing insight on this aspect of breeding phenology (e.g., red squirrels, *Tamiasciurus hudsonicus*—Lane et al., 2018, red deer, *Cervus elaphus*—Stopher et al., 2014, marmots, *Marmota flaviventris*—Ozgul et al., 2010, and Weddell seals, *Leptonychotes weddellii*—Rotella et al., 2016).

Grey seal (*Halichoerus grypus*) females are large, long‐lived (∼40 years) marine vertebrates, with an average post‐parturient body mass of about 200 kg. They are iteroparous, capital breeders that begin to reproduce at about age 5 and continue to reproduce annually over the course of several decades (Bowen, Iverson, Mcmillan, & Boness, 2006). Pregnant females exhibit a high degree of fidelity to breeding colonies and give birth to a single pup which they nurse for about 18 days (Bowen et al., 2015). Thus, individual females can be studied for many years. Females fast during lactation, so that nutrients supplied to offspring in the form of lipid‐rich milk and those required to meet maternal energy needs are derived entirely from body stores (Iverson, Bowen, Boness, & Oftedal, 1993). Weaning mass is a good measure of the energy invested in reproduction by female grey seals (Iverson et al., 1993), as it is positively correlated with juvenile survival in this species and is, therefore, a useful proxy of fitness (Hall et al., 2001; Bowen et al., 2015).

Here, we analyze 27 years of data from a well‐studied population of grey seals to investigate how maternal characteristics (age, parity, reproductive status in the previous year, and identity), demography (breeding colony density), and environmental conditions influence parturition dates and to assess the consequences of birthdate on offspring body mass at weaning. Our longitudinal data allowed us to estimate repeatability in parturition dates and assess the degree of individual plasticity of primiparous and multiparous females in response to environmental change.

Over the course of our study, decadal scale changes occurred in the physical and biological oceanography and in the fish and invertebrate communities in the continental shelf ecosystems (e.g., Shackell, Bundy, Nye, & Link, 2012) which constitute the main foraging areas of our population (Breed, Bowen, McMillan, & Leonard, 2006). These changes are thought to be partially driven by trends in several large‐scale climate indices, such as the North Atlantic Oscillation (NAO) and the Atlantic Multidecadal Oscillation (AMO) (Drinkwater et al., 2014), as well as from the effects of commercial fisheries (Shackell et al., 2012). The grey seal population has also increased continuously during our study (Hammill, den Heyer, Bowen, & Lang, 2017). Thus, we sought to understand the extent to which these large‐scale climate indices and trends in population size might explain the shift in mean birthdate in our study population and the consequences of that shift on offspring size at weaning, a good proxy for maternal energy investment in offspring.

## 2 MATERIALS AND METHODS

### 2.1 Study area and sampling

**Figure 1:**
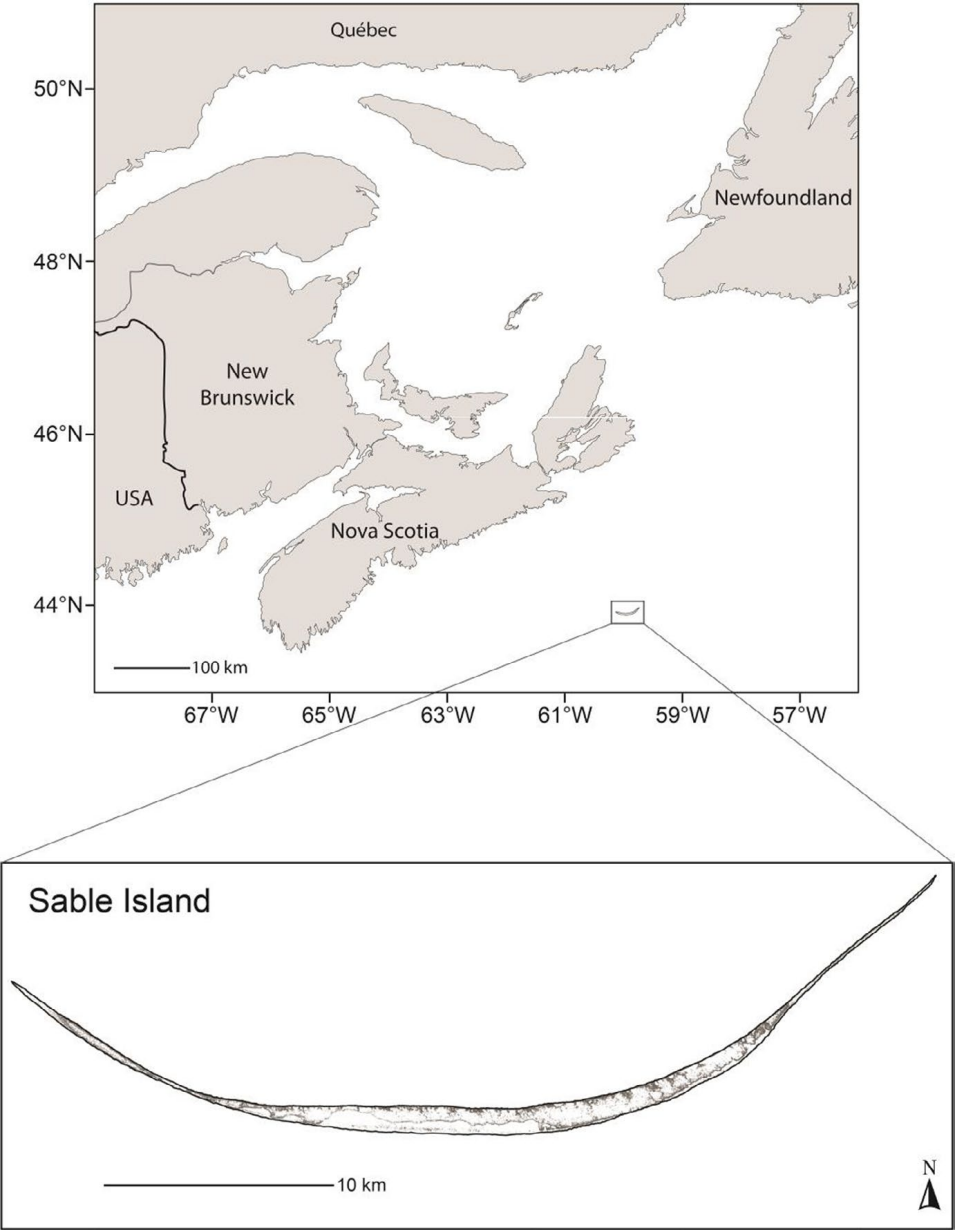
Map showing the location of the study colony

Our study was conducted on Sable Island (43°55′N, 60°00′W), Nova Scotia, Canada, during the December–February breeding season from 1991 to 2017. Located along the outer edge of the Scotian Shelf (Figure 1), Sable Island is a crescent shaped, partially vegetated sand bar approximately 42.5 km in length. Over the course of our study, the number of pups born annually increased some eightfold to 83,600 in 2016 (den Heyer, Lang, Bowen, & Hammill, 2017).

The known‐age adult females we studied are a subset of those that were marked with unique permanent hot‐iron brands shortly after weaning. A total of 3,387 female pups were uniquely branded in 1969, 1970, 1973, 1974, 1985–1987, 1989, and 1998–2002. The presence of a marked adult female in the breeding colony was determined from approximately weekly, whole‐island censuses conducted over the course of the breeding season each year (Bowen et al., 2006). At each observation, the female's geographic position in the colony, her reproductive status (with or without pup), and the pelage stage of her pup (an indication of pup age, Kovacs & Lavigne, 1985) were recorded.

Birthdates used for this study were known to within 24 hr. Although births were rarely observed, the day of birth could be determined reliably by the presence of birth fluids, blood, and placenta at the site, the presence of blood on the female's hind flippers, and the yellowish pelage of newly born pups. For the analysis, we transformed birthdate to days since December 1 of each breeding season (December 1 = day 0). Pups were marked with a semi‐permanent, uniquely numbered tag in the hind flipper and their sex was recorded. Study females and their pups were visited daily throughout lactation (but not disturbed) to obtain an accurate date of weaning, defined as the day a female left the pup and departed the colony. Pup weaning mass (to the nearest 0.5 kg) was measured on the day following the female's departure, and pup sex was confirmed.

The first year a female was observed either pregnant or with a pup was taken as the year of recruitment and used to calculate age of primiparity. Pregnant females have a clearly extended lower abdomen, visible bulge along one flank, and move in a characteristic rocking motion. A female's parity was defined as the number of breeding seasons that she was sighted pregnant or with a pup. As sighting probability is less than 1.0 (see below), and grey seals do breed elsewhere, it is possible that we could have missed the first birth of some females and, therefore, overestimated their age at first birth. However, about 85% of grey seal females in the Canadian seal population give birth at our study site (Hammill et al., 2017), so the impact of females breeding elsewhere on mean age of primiparity is expected to be small. Despite the objective of sighting all marked females on the island during the weekly censuses, some females are not sighted (Bowen et al., 2015). The estimated probability of sighting a female if she was alive on the island ranged from 0.8 to 0.95 between 1992 and 2016 (den Heyer & Bowen, 2017).

To determine reproductive success, females were classified as successful if they weaned a pup weighing > 35 kg or if the pup reached pelage stage ≥ 3 (i.e., late lactation) as pups at this stage have a high probability of survival (Bowen et al., 2015). Females were scored as unsuccessful if they weaned a pup < 35 kg, were not sighted, were only sighted pregnant, were only sighted with a pup early in lactation (before successful independence possible), or were observed with a dead pup.

### 2.2 Environmental data

**Figure 2:**
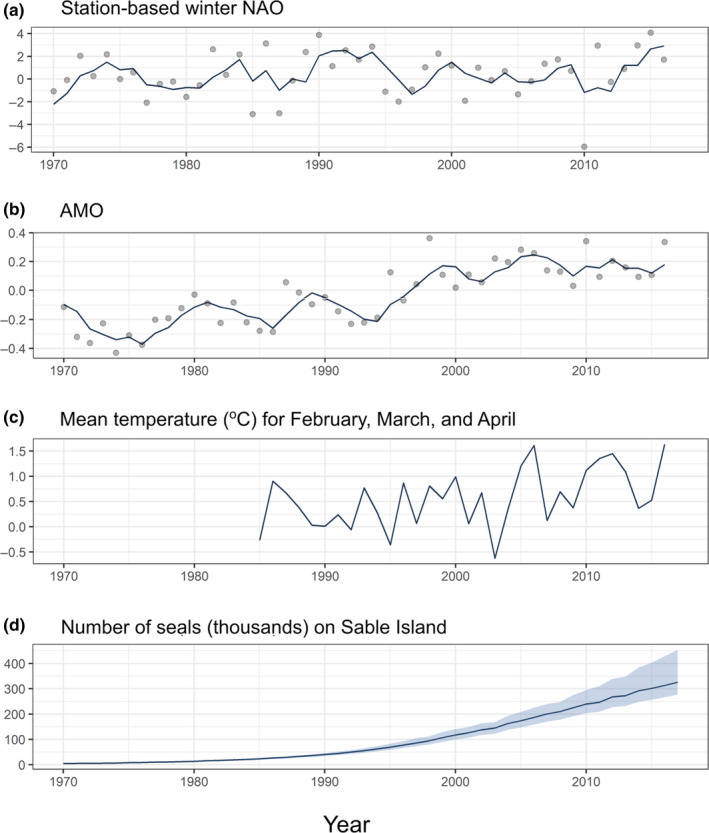
Long‐term trends in (a) the station‐based winter North Atlantic Oscillation (NAO), (b) the Atlantic Multidecadal Oscillation (AMO), (c) the annual mean surface water temperature for February, March, and April, and (d) number of seals (thousands) with 95% credible limits

Several environmental factors might influence birthdates of grey seals through effects on prey availability and, thus, the condition of pregnant females. The NAO is a large‐scale ocean‐atmosphere oscillation and an important driver of major water masses and currents in the Atlantic. Positive NAO corresponds to colder than normal temperatures over the Labrador‐Newfoundland Shelf, Gulf of St. Lawrence and Eastern Scotian Shelf and warmer conditions on the Central and Western Scotian Shelf and Gulf of Maine (Hebert, 2015). We used the station‐based index of the NAO obtained from Climate and Global Dynamics Laboratory (https://climatedataguide.ucar.edu/sites/default/files/nao_pc_djf.txt, downloaded on March 7, 2017). We tested the influence of the mean annual NAO index (Figure 2a) in the preceding year and preceding three years on birthdates. We chose a lag of three years to allow time for a change in the environment to be reflected the abundance of small fish prey that tend to dominate the diet of grey seals.

The AMO index describes multidecadal atmosphere and sea variability in the Atlantic (Trenberth & Zhang, 2016) with the warm phase associated with positive SST anomalies over most of the North Atlantic. We used the annual mean of AMO unsmoothed from the Kaplan SST V2 index calculated at NOAA/ESRL/PSD1 (http://www.esrl.noaa.gov/psd/data/timeseries/AMO/ downloaded from NOAA on March 12, 2017). The time series was detrended with 10‐year low‐pass filtered annual mean area‐averaged SST anomalies over the North Atlantic basin. Again, we tested the influence of the mean annual index (Figure 2b) in the preceding year and preceding 3 years on birthdates.

Coulson (1981) proposed that mean SST in the 3 months following mating largely determined parturition dates in grey seals by initiating the period of fetal growth. We obtained SST data on the Eastern Scotian Shelf (44°12′N to 45°40.2′N, and 60°00′W to 58°00′W), the main foraging area for adult female grey seals (Breed et al., 2006), for the months of February, March, and April. We used Advanced Very High Resolution Radiometer (AVHRR) Pathfinder Version 5.2 (PFV5.2, Casey, Brandon, Cornillon, & Evans, 2010) SST from 1985 to 2012 (Bedford Institute of Oceanography, Dartmouth, Nova Scotia, Canada). For 1998 to 2016, AVRR SST was downloaded from the NOAA and European Organization for the Exploration of Meteorological Satellites (EUMETSAT) database. A least‐square fit of the Pathfinder and NOAA temperatures during the 1991 to 2012 time period led to a conversion equation SST(NOAA) = 0.99062*SST(Pathfinder) + 0.16116 with an adjusted *r* ^2^ = 0.867. Using this regression, the Pathfinder data (1985–1997) were converted to be consistent with the more recent NOAA series (Figure 2c).

### 2.3 Population size

The increase in the size of the grey seal population since the 1960s could have increased competition for food which, in turn, could negatively impact maternal foraging success, body condition, and fetal growth and, therefore, birthdate in grey seals. Grey seal population size was estimated from an age‐structured population model fitted to a time series of pup production estimates by adjusting initial population size, adult mortality rate and carrying capacity (Hammill et al., 2017). In the model, density‐dependent changes in mortality are assumed to act on first year survival rate.

### 2.4 Statistical analysis

We estimated the repeatability of parturition dates of females as the proportion of the total variation attributed to variation among individuals compared to variation among measurements within individuals with ICC package in R based upon the exact confidence limit equation in Searle (1971), which can be used for unbalanced data (Wolak, Fairbairn, & Paulsen, 2012).

We used generalized linear mixed models (GLMM) to examine relationships between pup birthdate and covariates (intrinsic and extrinsic) and the consequences of variation in birthdate on pup body mass at weaning. Models were fit using the lme4 package in R (Bates, Mächler, Bolker, & Walker, 2015), and model selection was based on Akaike information criterion (AIC_c_), with smallest ΔAIC, and highest AIC weights (*w*) (Burnham & Anderson, 2002) being preferred models, using the Multi‐Model Inference Package (Barton 2016). We used the methods of Nakagawa and Schielzeth (2013) to estimate marginal *R* ^2^ ($R_{\text{GLMM(m)}}^{2}$), representing the variance explained by fixed factors, and conditional *R* ^2^ ($R_{\text{GLMM(c)}}^{2}$), describing the total variance explained by the models. For model selection, all continuous covariates were standardize to a mean of zero and divided by the standard deviation. To estimate model coefficients, the preferred models were run with raw covariates. The error estimates of fixed and random effects were reconstructed from the bootstrapped confidence interval (nsim = 1,000) assuming the interval is normally distributed (Duursma, 2019) and plotted using produced using visreg package (Breheny & Burchett, 2017). All statistical analyses were conducted using R 3.6.1 (R Core Team, 2019).

Maternal traits used as covariates in the model included maternal age, parity, and reproductive success in the previous year. We modeled the effect of maternal age as a quadratic as our previous research has shown that other reproductive traits vary in this way (Bowen et al., 2006). Grey seals begin to reproduce at about 5 years of age and continue for several decades (Bowen et al., 2006). Therefore, we modeled parity as a factor (1, 2, 3, 4, 5+) to accommodate change as females gained reproductive experience, but we expected little change in females that had given birth 5 or more times. A female's reproductive success in 1 year can influence her success in a subsequent year (e.g., Hadley, Rotella, & Garrott, 2007); therefore, reproductive success in the previous year was included as a factor (0 = unsuccessful, 1 = successful). Pup sex (factor: 1 = male, 2 = female) affects several offspring traits in grey seals (Bowen et al., 2006) and other phocid seal species and therefore was also included in the model. Study year was included as a factor to allow for nonlinear environmental variation. The above covariates were treated as fixed effects in the full model of birthdate. Post hoc analysis was used to reduce the number of levels to describe the influence of parity on birthdate.

We used a 3‐step process to develop a model that includes interactions between intrinsic and extrinsic covariates. In the first step, we develop a mixed model that predicts parturition dates as a function of intrinsic covariates and environmental variability described by year as a factor, while addressing the individual heterogeneity with the random effect. Next, we ask which environmental indices best describe the mean parturition date each year as estimated from first model. In the third step, we include in the parturition date model the intrinsic covariates identified in the first step, the environmental covariates identified in the second step, as well as interactions between the environmental covariates and parity.

Birthdate, *B_ij_* (i.e., female *i, pup j*) was modeled as an independent random normal variable (*ε_ij_* = *N*(0, *σ* ^2^). To account for the individual variation in parturition dates, female identity was included as a random effect (ϒ*_i_* = *N*(0, *d* ^2^). The full model was as follows:

$$\begin{array}{c} B_{\mathrm{ij}}=\beta_{\text{parity}}\times {\;\text{parity}}_{\mathrm{ij}}+\beta_{\text{sex}}\times {\text{Malepup}}_{\mathrm{ij}}+\beta_{\text{suc}}\times {\;\text{previous success}}_{\mathrm{ij}}+\beta_{\text{age}}\times {\text{maternal age}}_{\mathrm{ij}}\\ +\beta_{\text{age}2}\times I\left({\text{maternal age}}_{\mathrm{ij}}^{2}\right)+\beta_{\text{year}}\times {\text{year}}_{\mathrm{ij}}+\Upsilon_{i\;+}\varepsilon_{\mathrm{ij}}. \end{array}$$

Post hoc tests were used to select the best model among those with a different number of parity bins.

To identify the best model to predict mean annual birthdate estimated from the previous model (*β*_year_ *_i_*), time‐varying environmental and population covariates (NAO, AMO, SST, and number of pups) and interactions were included in multiple linear regression. Where

$$\beta_{\text{year}\;i}=\beta_{\text{amo}}\times {\text{amo}}_{i}+\beta_{\text{amo3yr}}\times {\text{amo3yr}}_{I}+\beta_{\text{nao}}\times {\text{nao}}_{i}+\beta_{\text{nao3yr}}\times {\text{nao3yr}}_{I}+\beta_{\text{SST}}\times {\text{SST}}_{i}+\beta_{\text{popsize}}\times {\text{popsize}}_{i}+\alpha +\varepsilon_{i}.$$

For NAO and AMO, the previous year and previous 3‐year mean were included in model selection but prohibited from being in the same model as they were highly correlated. For the same reason, the AMO 3‐year mean and population size were not used in the same model. The covariates from the preferred model of mean birthdate were subsequently included in the model with intrinsic effects to explore interactions between environmental covariates and parity:

$$\begin{array}{c} B_{\mathrm{ij}}=\beta_{\text{parity}}\times {\text{parity}}_{\mathrm{ij}}+\beta_{\text{sex}}\times {\text{Malepup}}_{\mathrm{ij}}+\beta_{\text{suc}}\times {\text{previous success}}_{\mathrm{ij}}\\ +\beta_{\text{age}}\times {\text{maternal age}}_{\mathrm{ij}}+\beta_{\text{age}2}\times \;I\left({\text{maternal age}}_{\mathrm{ij}}^{2}\right)\\ +\beta_{\text{popsize}}\times {\text{Popsize}}_{\mathrm{ij}}+\beta_{\text{amo}}\times {\text{amo}}_{\mathrm{ij}}+\beta_{\text{nao}3\text{yr}}\times \text{nao}3{\text{yr}}_{\mathrm{ij}}\\ +\beta_{\text{popsize:parity}}\times {\text{Popsize}}_{\mathrm{ij}}\times {\text{parity}}_{\mathrm{ij}}+\beta_{\text{amo:parity}}\times {\text{amo}}_{\mathrm{ij}}\\ \times {\text{parity}}_{\mathrm{ij}}+\beta_{\text{nao}3\text{yr}:\text{parity}}\times \text{nao}3{\text{yr}}_{\mathrm{ij}}\times {\text{parity}}_{\mathrm{ij}}+\Upsilon_{i}+\varepsilon_{\mathrm{ij}}. \end{array}$$

Finally, to examine the consequences of a shift in birthdate on reproductive performance of females, we fitted GLMM models to the mass of pups at weaning (*M_ij_*) (Gaussian), again with female identity as random effect (ϒ*_i_* = *N*(0, *d* ^2^). All pups regardless of body mass were used in this analysis. Parity (factor: 1, 2, 3, 4, and 5+), success in the previous year (factor: 0, 1), pup sex (factor: 1, 2), a quadratic of maternal age the birthdate anomaly within the breeding season (i.e., difference in days from mean birthdate each year), and the mean birthdate for the breeding season:

$$\begin{array}{c} M_{\mathrm{ij}}=\beta_{\text{parity}}\times {\text{parity}}_{\mathrm{ij}}+\beta_{\text{sex}}\times {\text{Malepup}}_{\mathrm{ij}}+\beta_{\text{suc}}\times {\text{previous success}}_{\mathrm{ij}}\\ +\beta_{\text{age}}\times {\text{maternal age}}_{\mathrm{ij}}+\beta_{\text{age}2}\times I\left({\text{maternal age}}_{\mathrm{ij}}^{2}\right)\\ +\beta_{\text{bdanomaly}}\times {\text{bdanomaly}}_{\mathrm{ij}}+\;\beta_{\text{meanbd}}\times {\text{meanbd}}_{\mathrm{ij}}+\Upsilon_{i}+\varepsilon_{\mathrm{ij}}. \end{array}$$

## 3 RESULTS

Over the course of our 27‐year study, we recorded birthdates for 2,768 pups of 660 females from 13 marked cohorts (S1). The mean number of birthdates per year was 102 (range = 24 to 178). Fewer than 50 birthdates were recorded in only 5 of the 27 years. The number of parturition dates for individual females ranged from 1 to 19, and half of the females had ≥3 pups with known birthdate (S2). The overall average age of females in the study was 16 years (*SD* = 7.9, range 4–41 years). Of the pups studied, sex was not available for 66 pups usually because the female had left the colony (i.e., abandoned) before the pup could be sexed and tagged. The proportion of female pups born over the course of the study was 0.51 (95% CI = 0.48–0.53, *n* = 1,365) and of males was 0.49 (95% CI = 0.48–0.51, *n* = 1,337). Thus, sex ratio did not depart from 1:1.

In the 499 females with more than one parturition date, the intraclass correlation (ICC) was 0.66 (95% CI = 0.63–0.70), indicating that parturition dates of individual females were highly repeatable.

### 3.1 Factors influencing birthdate

Year had the strongest influence on the mean birthdate of all the covariates in the preferred model (Table 1, Figure 4e). The fixed effects in the model with only intrinsic factors explained 8% of the variance ($R_{\text{GLMM(m)}}^{2}$ = 0.08, $R_{\text{GLMM(c)}}^{2}$ = 0.84), while the fixed effects in the model that included both intrinsic factors and year explained 28% of the variance ($R_{\text{GLMM(m)}}^{2}$ = 0.28, $R_{\text{GLMM(c)}}^{2}$ = 0.84). Birthdates advanced by more than 2 weeks over the 27 years of the study, with the greatest change occurring in the late 1990s (Figure 4e). Mean birthdate varied from January 12 (day 42) to January 15 (day 45) until about 1995 and then advanced to January 4 (day 34) by 2000. There appeared to have been a brief period of relative stasis around 2005 and then a further decrease through the end of the study with mean birthdate between December 24 to 26 in recent years (day 25 and day 27).

**Table 1:** Model selection for the mixed‐effect models of pup birthdate, including female parity (*β*_parity_), pupping success in the previous year (*β*_suc_), and maternal age (*β*_age_ + *β*_age2_) which was normalized and included as a quadratic, pup sex (*β*_sex_), year as a factor (1991–2017) and seal identity as a random effect (*ϒ*_j_)

Model	*K*	LL	AIC_c_	ΔAIC_c_	*w_i_*
*β*_parity_ × parity*_ij_* + *β*_sex_ × Malepup*_ij_* + *β*_suc_ × previous success*_ij_* + *β*_age_ × maternal age*_ij_* + *β*_age_ *_2_* × I (maternal ${\text{age}}_{\mathrm{ij}}^{2}$) + *β*_year_ × year*_ij_* + *ϒ_j_*	37	−7723.4	15,521.9	0	0.998
*β*_parity_ × parity*_ij_* + *β*_sex_ × Malepup*_ij_* + *β*_age_ × maternal age*_ij_* + *β*_age2_ × I(maternal ${\text{age}}_{\mathrm{ij}}^{2}$) + *β*_year_ × year*_ij_* + *ϒ_j_*	36	−7730.7	15,534.5	12.6	0.002
*β*_parity_ × parity*_ij_* + *β*_suc_ × previous success*_ij_* + *β*_age_ × maternal age*_ij_* + *β*_age2_ × I (maternal ${\text{age}}_{\mathrm{ij}}^{2}$) + *β*_year_ × year*_ij_ + ϒ_j_*	36	−7737.2	15,547.5	25.6	<0.001
*β*_parity_ × parity*_ij_* + *β*_age_ × maternal age*_ij_* + *β*_age2_ × I(maternal ${\text{age}}_{\mathrm{ij}}^{2}$) + *β*_year_ × year*_ij_* + *ϒ_j_*	35	−7745.4	15,561.8	39.9	<0.001
*β*_sex_ × Malepup*_ij_* + *β*_suc_ × previous success*_ij_* + *β*_age_ × maternal age*_ij_* + *β*_age2_ × I (maternal ${\text{age}}_{\mathrm{ij}}^{2}$) + *β*_year_ × year*_ij_* + *ϒ_j_*	33	−7787.0	15,640.8	118.9	<0.001

Note

The five models with the lowest AIC are reported. *N*_seals_ = 654, *N*_obs_ = 2,702.

Abbreviations: AICc, Akaike information criterion for small sample sizes; K, number of parameters; LL, Log likelihood; wi, AIC weights; ΔAICc, relative change in AICc.

**Figure 3:**
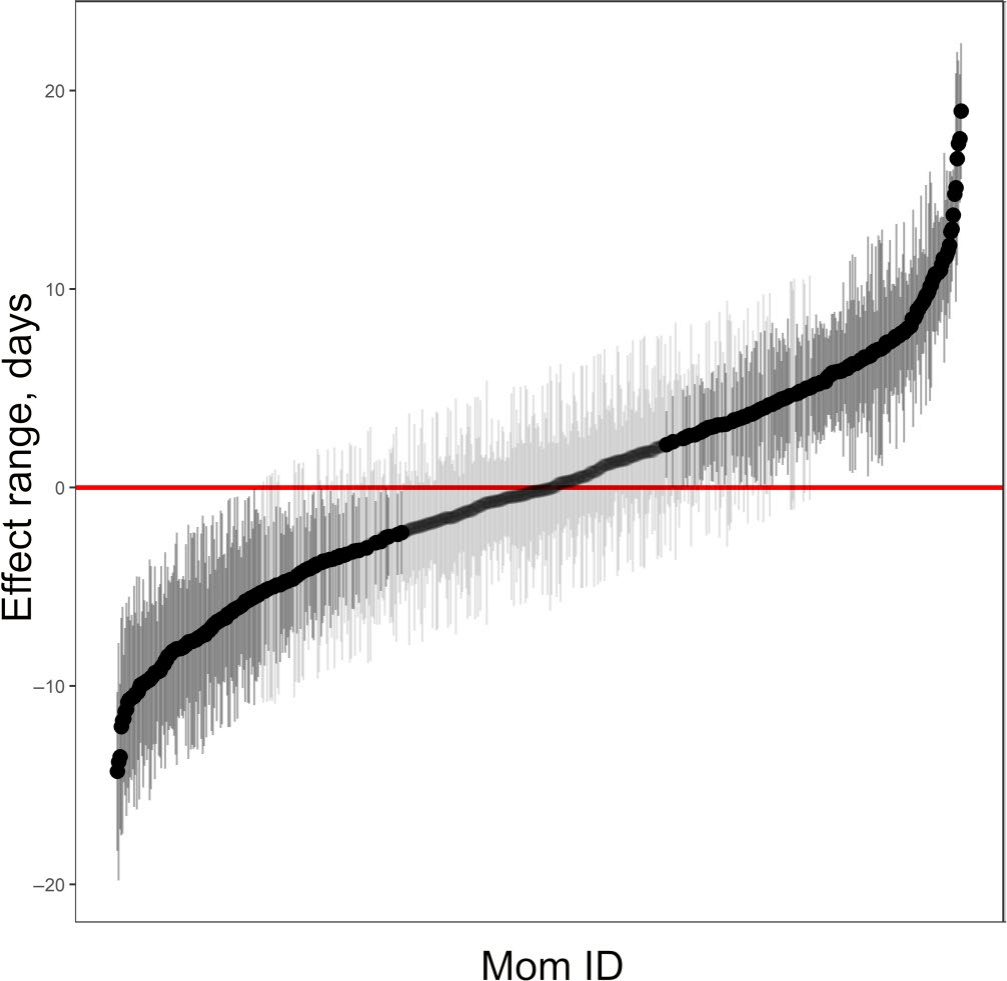
Plot of the simulated random effects of individual grey seal females (MomID) during a 27‐year study (1991–2017) at Sable Island, Nova Scotia from the mixed‐effects model of pup birthdate. Black dots represent deviation in days from the model intercept for the 654 individual females. The darker shaded whiskers (95% confidence regions) are those which do not cross 0, the model intercept (red line)

**Figure 4:**
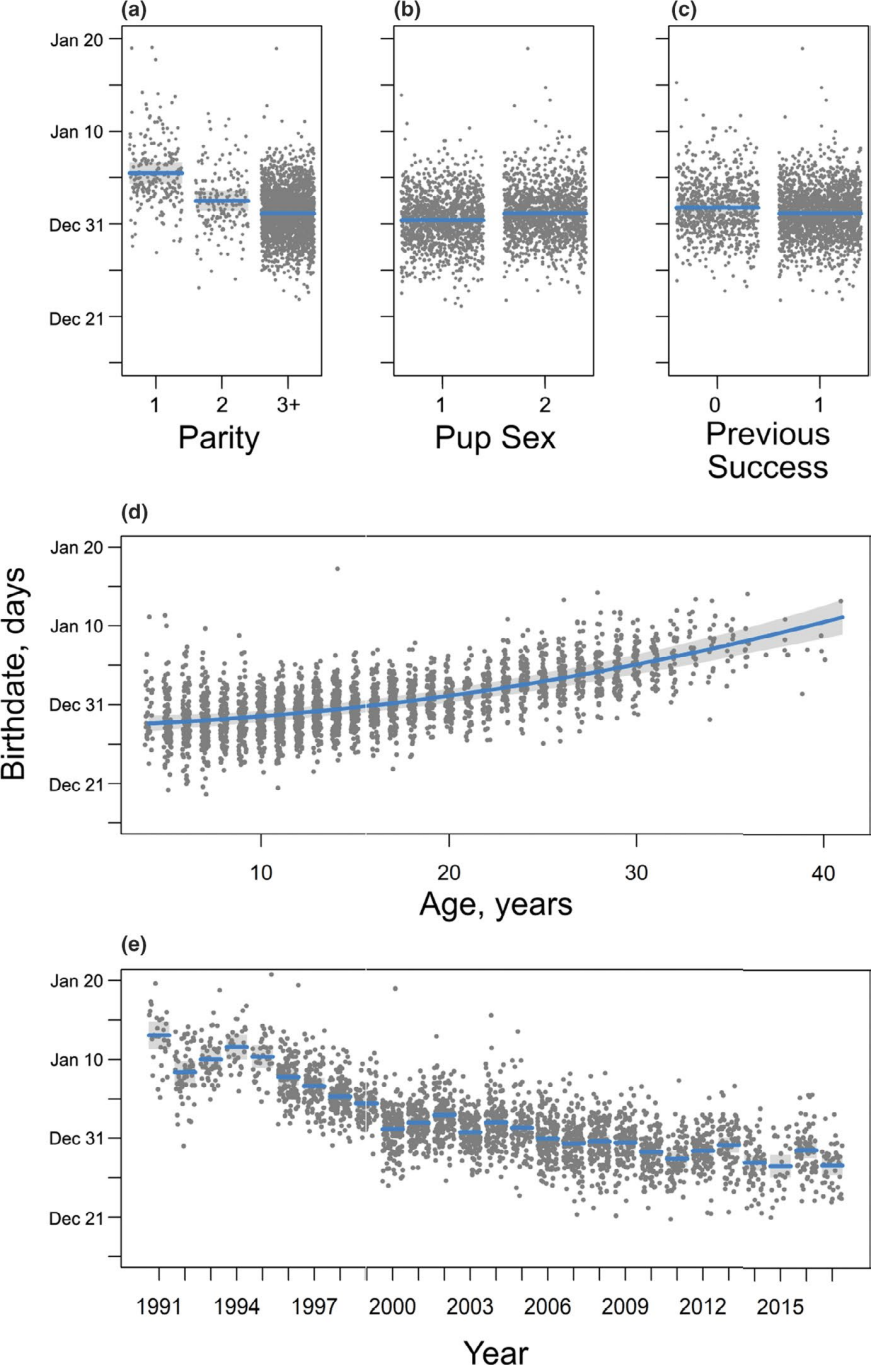
Plot of birthdates of individual grey seal pups (grey dots) during a 27‐year study (1991–2017) at Sable Island, Nova Scotia. Each panel shows the prediction (blue line) and 95% bootstrapped confidence interval (light grey band) for birthdates from the mixed model: (a) parity, (b) pup sex, (c) pupping success in previous year (Previous Success), (d) maternal age (Age), and (e) year as a factor. Conditional plots are presented for a mother which had breeding success in previous (Previous Success = 1) had 2 or more pups (Parity = 3+), a female pup, in year 2000, and was 20 years old

All of the other intrinsic covariates (parity, success in the previous year, pup sex, and maternal age) were retained in the maximal model of birthdate (Table 1). Coefficients from the preferred model are given in S3. Variation in birthdate among females was the largest source of variation in the model, reflecting the high repeatability in birthdates of individual females (Figure 3). Post hoc analysis of parity indicated that three bins (i.e., primiparous females, second‐time breeders, and experienced breeders with ≥3 pups) were marginally better than a model with two or four parity bins (S4). Of the intrinsic fixed effects in the preferred model, parity had the greatest influence on birthdate. Primiparous grey seal females gave birth three days later than second‐time breeders and four days later than multiparous females with three or more previous offspring (Figure 4a). Pup sex also influenced birthdate such that female pups were born about 0.7 days later than male pups (Figure 4b). Females that successfully weaned a pup in the previous year gave birth 0.6 days earlier than those that had not (Figure 4c). On average, birthdates predicted from the model occurred later in the breeding season as females got older, although there was considerable variability in birthdates for a given age (Figure 4d).

### 3.2 Influence of environmental covariates

**Figure 5:**
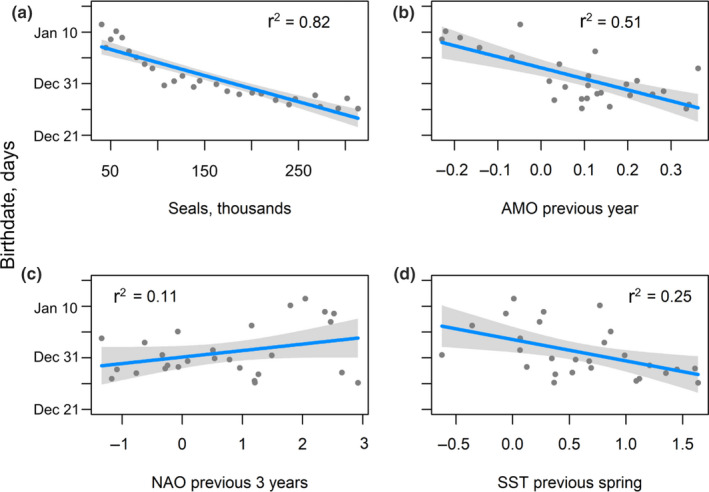
Predicted mean birthdate (grey dots) of grey seal pups during a 27‐year study (1991–2017) at Sable Island, Nova Scotia plotted against the (a) number of pups produced on Sable Island, (b) the Atlantic Multidecadal Oscillation (AMO) in the previous year, (c) the North Atlantic Oscillation (NAO) in the previous 3 years, and (d) the sea surface temperature (SST) in the previous spring. The shaded areas are partial residuals. The correlation coefficient (*r* ^2^) is reported in each plot

Although year as a factor explained much of the temporal variability in mean birthdates (Figure 4e), it provided little information about the nature of the environmental forcing that may underlie the observed shift in birthdates over time. The predicted mean birthdate was negatively correlated with the total population size, the AMO in the previous year, and annual mean SST over the 3 months preceding implantation (February‐April), and positively correlated with the mean NAO in the previous 3 years (Figure 5). The preferred model to describe mean birthdate included population size, AMO and the NAO in the 3 years before the breeding season (Table 2).

**Table 2:** Model selection for the multiple linear regression of mean pup birthdate relative to December 1, including AMO (*β*_AMO_), 3‐year mean AMO (*β*_AMO3yr_), NAO (*β*_NAO3_), 3‐year mean NAO (*β*_NAO3yr_), SST (*β*_SST_), and intercept (α)

Model	*K*	LL	AIC_c_	ΔAIC_c_	*w_i_*
*β*_amo_ × amo*_i_* + *β*_nao3yr_ × nao3yr*_I_* + *β*_popsize_ × Popsize*_i_* + α	5	−52.27	117.39	0	0.6032
*β*_amo_ × amo*_i_* + *β*_nao_ × nao*_i_* + *β*_popsize_ × Popsize*_i_* + α	5	−53.20	119.26	1.87	0.2364
*β*_amo_ × amo*_i_* + *β*_nao3yr_ × nao3yr _I_ + β_SST_ × SST*_i_* + *β*_popsize_ × Popsize*_i_* + α	6	−52.27	120.73	3.34	0.1135
*β*_amo_ × amo*_i_* + *β*_nao_ × nao*_i_* + *β*_SST_ × SST*_i_* + *β*_popsize_ × Popsize*_i_* + α	6	−53.20	122.61	5.22	0.0444
*β*_amo3yr_ × amo3yr_I_ + *β*_nao3yr_ × nao3yr_I_ + α	4	−60.03	129.87	12.48	0.0011

Note

The five models with the lowest AIC are reported. *N* = 27.

K, number of parameters; AICc, Akaike information criterion for small sample sizes; ΔAICc, relative change in AICc; and wi, AIC weights

**Figure 6:**
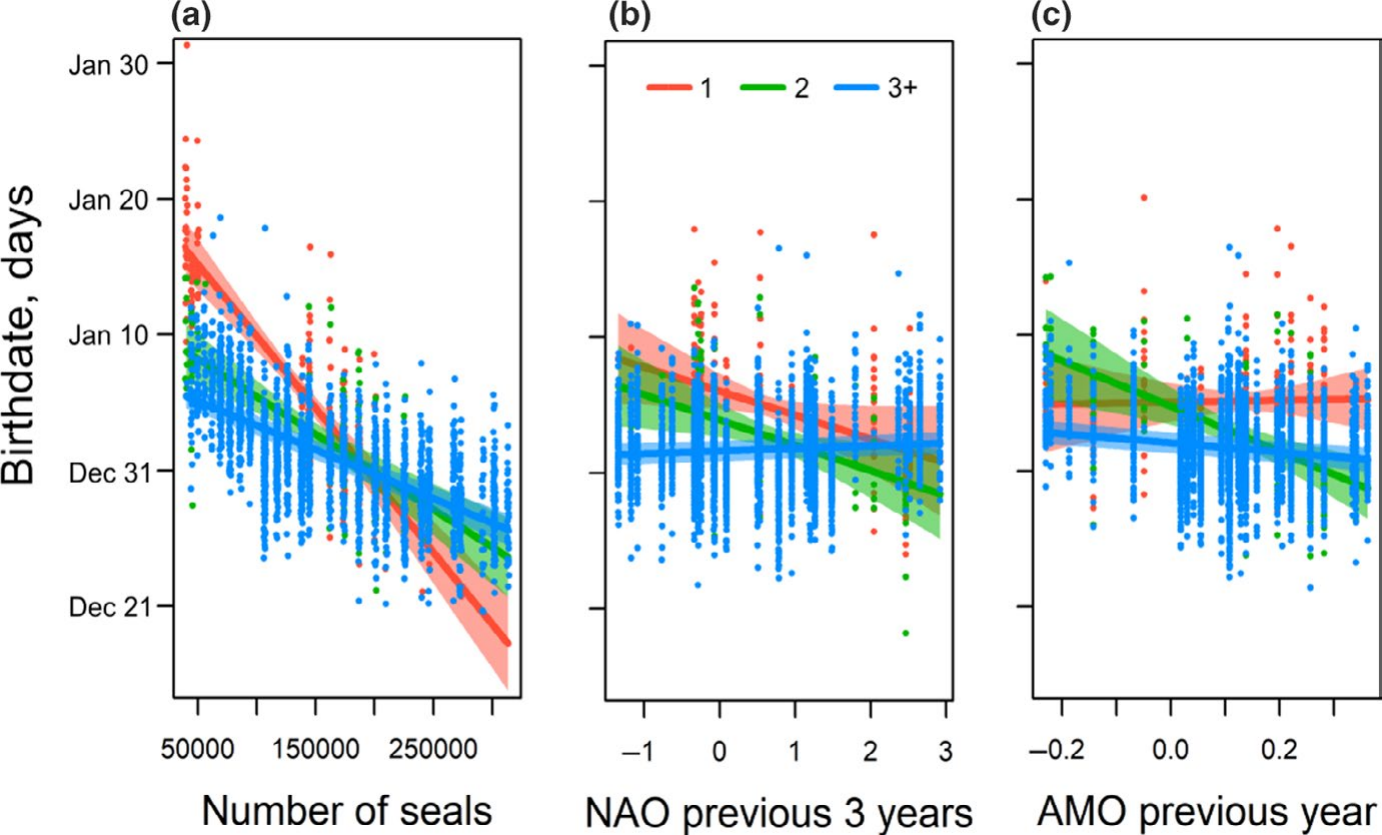
Plot of birthdates of grey seal pups during a 27‐year study (1991–2017) at Sable Island, Nova Scotia overlaid with the prediction (solid line) and bootstrapped 95% confidence intervals (colored shaded areas) of birthdate for parity 1 (red), parity 2 (green), and parity 3+ (blue) females from the mixed model including intrinsic factors and (a) Population size, (b) North Atlantic Oscillation (NAO) in the previous three years, and (c) Atlantic Multidecadal Oscillation (AMO) in the previous year. Conditional plots are based on a mother which had produced a successful pup in the previous year (Previous Success = 1), had 2 or more pups previously (Parity = 3+), had a female pup in year 2000, was 20 years old, and the mean of the other continuous fixed effects

To explore more specific environmental influences on birthdates, we dropped year from the model and added the environmental time series as a fixed effect with interactions between parity and the AMO in the previous year and population size (Table 3). The preferred model included interactions between parity and environmental covariates, which is evidence for heterogeneity in the response of females differing in reproductive experience to environmental forcing (Table 3, Figure 6). For example, primiparous female birthdates exhibited a stronger negative relationship with population size than multiparous females (Figure 6a). Again, variation in birthdate among females was the largest source of variation in the model, reflecting the high repeatability in individual birthdates (S5). The preferred model explained 80% of the variance in birthdate ($R_{\text{GLMM(c)}}^{2}$ = 0.80), with the fixed effects explaining one‐fifth of the variance ($R_{\text{GLMM(m)}}^{2}$ = 0.21).

**Table 3:** Model selection for the mixed‐effect models pup birthdate

Environmental covariates	*K*	LL	AIC_c_	ΔAIC_c_	*w_i_*
+*β*_popsize_ × Popsize*_ij_* + *β*_amo_ × amo*_ij_* + *β*_nao3yr_ × nao3yr*_ij_ + β*_popsize:parity_ × Popsize*_ij_* × parity*_ij_* + *β*_amo:parity_ × amo*_ij_* × parity*_ij_* + *β*_nao3yr:parity_ × nao3yr*_ij_* × parity*_ij_*	18	−7927.64	15,891.54	0	0.9100
+*β*_popsize_ × Popsize*_ij_ *+ *β*_amo_ × amo*_ij_* + *β*_nao3yr_ × nao3yr*_ij_* + *β*_popsize:parity_ × Popsize*_ij_* × parity*_ij_* + *β*_nao3yr:parity_ × nao3yr*_ij_* × parity*_ij_*	16	−7932.19	15,896.59	5.0496	0.0729
+*β*_popsize_ × *Popsize*_ij_ + *β*_amo_ × amo*_ij_* + *β*_nao3yr_ × nao3yr*_ij_* + *β*_popsize:parity_ × *Popsize*_ij_ × parity*_ij_* + *β*_amo:parity_ × amo*_ij_* × parity*_ij_*	16	−7933.99	15,900.18	8.6412	0.0121
+*β*_popsize_ × Popsize*_ij_* + *β*_amo_ × *amo*_ij_ + *β*_popsize:parity_ × Popsize*_ij_* × parity*_ij_* + *β*_amo:parity_ × amo*_ij_* × parity*_ij_*	15	−7935.91	15,902	10.4596	0.0049

Note

The top 4 models included the intrinsic variable: female parity (*β*_parity_), pupping success in the previous year (*β*_suc_), and maternal age (*β*_age_ + *β*_age2_) which was normalized and included as a quadratic, the sex of the pup sex (*β*_sex_), and individual seal as a random effect (*ϒ_j_*). Model selection identified the preferred environmental covariates, total population mean AMO (*β*_AMO3yr_), 3 year mean NAO (*β*_NAO3yr_), and the interaction between environmental covariates and parity. The five models with the lowest AIC are reported. *N*_seals_ = 654, *N*_obs_ = 2,702.

K, number of parameters; AICc, Akaike information criterion for small sample sizes; ΔAICc, relative change in AICc; and wi, AIC weights

### 3.3 Consequences of temporal trend in birthdate

**Figure 7:**
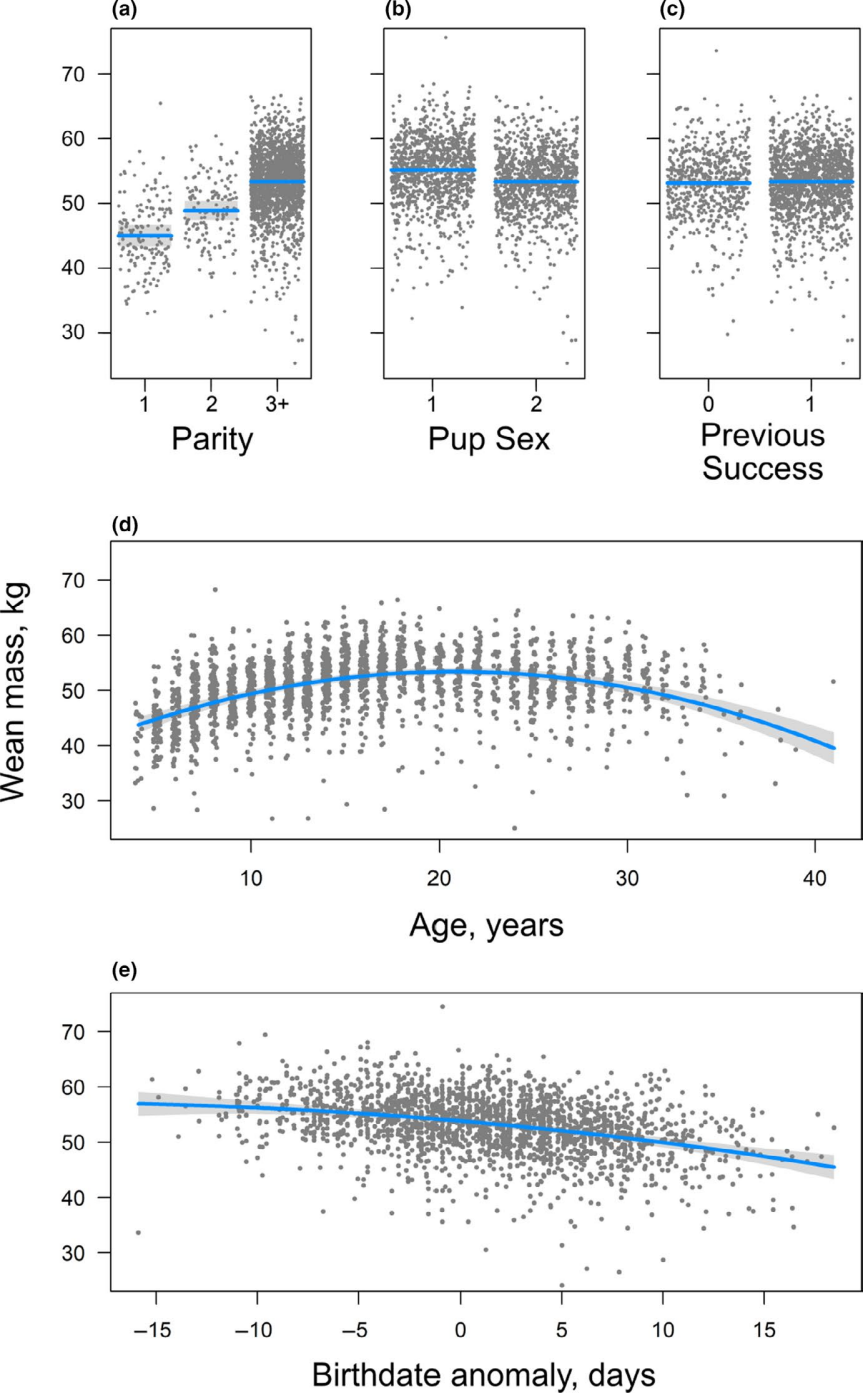
Plot of pup weaning mass in kg (grey dots); each panel shows the prediction (blue line) and 95% bootstrapped confidence interval (light grey band) for birthdates from the mixed model: (a) parity, (b) pup sex, (c) pupping success in previous year (Previous Success), (d) maternal age (Age), and (e) birthdate anomaly. Conditional plots are presented for a mother which had breeding success in previous year (Previous Success = 1), had 2 or more pups previously (Parity = 3+), had a female pup in year 2000, was 20 years old and born in the middle of the breeding season

To estimate the consequences of variation in birthdate on offspring weaning mass, we tested a model which included maternal age, parity, reproductive success in the year previous, pup sex, and birthdate anomaly as fixed effects and female identity as a random effect. Each of these fixed effects has previously been shown to influence pup weaning mass in this species (Boness et al., 1995; Bowen et al., 2006; Weitzman, den Heyer, & Bowen, 2017). The preferred model explained 58% of the variance ($R_{\text{GLMM(c)}}^{2}$ = 0.58), with the fixed effects explaining roughly half ($R_{\text{GLMM(m)}}^{2}$ = 0.29). Pup weaning mass was influenced by pup sex, parity, female age, and birthdate anomaly (Table 4, Table S6) with females weighing about 2 kg less than males. Pups born later in the breeding season had lower weaning mass (Figure 7), but the mean birthdate for a given year did not explain additional variation in pup weaning mass (Table 4). Thus, we found no evidence that the change in phenology of the breeding colony influenced pup weaning mass.

**Table 4:** Parameter estimates from the mixed‐effect model of pup weaning mass, including female parity (Parity), pupping success in the previous year (Successy‐1), maternal age (Age) which was normalized and included as a quadratic, pup sex (Pup Sex), and the birthdate anomaly for that pup

Coefficients	Estimate	*SE*	*t* value
(Intercept)	45.4	45.38	0.78
Parity 2	3.9	3.86	0.79
Parity 3+	8.4	8.37	0.70
Sex (Female)	−1.8	−1.79	0.27
Previous success	0.24	0.24	0.29
Age	2.4	2.40	0.26
I (Age)^2	−2.1	−2.11	0.17
Birthdate anomaly	−2.2	−2.17	0.22

Note

Parity 2 = second parity females, Parity 3+ = females with 3 or more pups. *N*_obs_ = 2,135, *N*_seals_ = 600.

## 4 DISCUSSION

Over the 27 years of our study, mean birthdate of grey seal pups advanced by 15 days. This represents one of the largest and most rapid phenological shifts that has been observed in long‐term studies of vertebrates (Parmesan, 2006; Thackeray et al., 2010). The shift in birthdate was correlated with an increase in population size, the AMO, and to a lesser extent the NAO which was averaged over the previous 3 years suggesting that a large‐scale environment forcing was largely responsible. Our longitudinal data enabled us to assess the impact of environmental changes on both first time and experienced breeders. Females generally responded to the environmental change by advancing parturition dates, but there was evidence for heterogeneity in the response of individuals. Presumably, because they were in better condition, multiparous females gave birth earlier and their birthdates shifted less over time than those of primiparous females. Primiparous females entering the breeding population in the early 2000s gave birth almost 2 weeks earlier than those in the 1990s. Despite the large temporal shift to earlier birthdates, we found no evidence of a change in maternal reproductive performance as measured by the body mass of offspring at weaning (a good predictor of recruitment to the breeding population, Bowen et al., 2015) in this capital breeding species.

### 4.1 Intrinsic and extrinsic effects on birthdate

Breeding phenology is influenced by both intrinsic and extrinsic factors and their interactions across diverse taxa (Stopher et al., 2014). However, few studies have examined the simultaneous influence of multiple factors on the timing of births. We found that maternal identity, age, parity, and success in the previous year all influenced birthdates in grey seals, but maternal identity and parity had the largest effects. In grey seals, primiparous females gave birth later in the season than multiparous females (Figure 7). This contrasts strongly with the earlier parturition date of less‐experienced northern elephant seal (*Mirounga angustirostris*) females, where earlier breeding may have the advantage of reducing the negative effects associated with the increased colony density later in the breeding season (Sydeman, Huber, Emslie, Ribic, & Nur, 1991). Other factors must underlie the later breeding of less‐experienced grey seal females as later breeding females experience increasing density and potentially greater disturbance (Boness et al., 1995).

Females that raised offspring in the previous year were predicted to give birth later than females which did not because they might have reduced body condition and, therefore, would have fewer resources to allocate to fetal growth. However, success in the previous year had weak effects on birthdate in grey seals (this study) and Weddell seals (Rotella et al., 2016). Given the small observed effect size, it is difficult to assess whether the effect is biologically significant in these species. Nevertheless, our results are consistent with findings in red deer (Clutton‐Brock, Guinness, & Albon, 1983), and Bighorn sheep (*Orvis canadensis*, Feder, Martin, Festa‐Bianchet, Bérubé, & Jorgenson, 2008) suggesting that previous reproductive performance generally has a modest influence on birth date in many mammals.

It could be argued that the earlier birth of males compared to females may be a consequence of the greater role that body size plays in the fitness of males relative to that of females in sexual dimorphic species. In Weddell seals, male pups were born about two days earlier than female pups (Rotella et al., 2016). Although the effect was not as strong in the present study, we similarly found that male grey seal pups were born earlier than female pups as previously reported by Coulson and Hickling (1963). There are conflicting results in other pinnipeds (Boltnev & York, 2001; Bowen, Oftedal, Boness, & Iverson, 1994) and other mammals (Cote & Festa‐Bianchet, 2001) where males are not born earlier than females suggesting that the sex of offspring may generally have little influence on birthdate.

Seasonal breeding is widespread among large mammals and is thought to evolve to allow births to occur at a time when food and climate are favorable for reproductive success (Bronson, 1989). Year had the greatest influence on mean birthdate in grey seals, explaining some 20% of the observed variation. Interannual variation in mean birthdate at our study site could indicate that annual changes in the ocean conditions influenced the temporal distribution of births. Such interannual variation on maternal body condition, mediated through weather and food supply, is evident in both terrestrial and other marine species (Bowyer, 1991; Boyd, 1984; Brommer, Rattiste, & Wilson, 2008; Keech et al., 2000; Nussey, Clutton‐Brock, Albon, et al., 2005; Nussey, Clutton‐brock, Elston, et al., 2005; Przybylo et al., 2000; Rotella et al., 2016). In female pinnipeds, physiological condition and the storage of energy to support reproduction are unlikely to depend on the direct effects of weather, but rather are more likely to be manifested through changes in food availability. Thus, we looked for associations between large‐scale climate drivers that are known to have effects on marine species. We found that the predicted mean birthdate was negatively correlated with the mean AMO in the previous year, and weakly positively correlated to the mean NAO in the previous 3 years (Figure 5). However, the seal population size was most strongly correlated to the birthdate timing (Table 2). Since the beginning of our study, the AMO index has exhibited a warming trend. Alheit et al. (2014) showed that the dynamics of abundance and migrations of populations of small pelagic clupeoid fishes in the eastern north and central Atlantic vary in synchrony with the warm and cool phases of the AMO, with increasing abundance during the warm phase of the index. More generally, Drinkwater et al. (2014) identified biological impacts of the AMO in the northern North Atlantic to include a general increase in plankton and fish productivity, as well as expansion of the species distributions northward, in conjunction with warm periods and the opposite during cold periods. Shackell et al. (2012) examined temporal changes in ecosystem structure on the Scotian shelf, the main foraging grounds of female grey seals (Breed et al., 2006), and the adjacent large ecosystems in which these animals forage. They found a steady increase starting in the 1980s in the abundance of phytoplankton, the biomass of decapods and planktivores, the biomass of a noncommercial fish, the biomass of medium benthivores, and, to a lesser extent, the biomass of zoopiscivores. Grey seals are generalist piscivores known to feed on a variety of small pelagic and demersal fishes (Bowen & Harrison, 1994). These changes suggest that there may have been an increase in the abundance of prey available to greys seals. This is further supported by the monotonic increase in grey seal population size at our study site during the period of our study (Hammill et al., 2017). Thus, we interpret the high correlation between the AMO and mean birthdate as a large‐scale causal mechanism likely responsible for an increase in prey available to grey seal females. If our hypothesis is correct, the return to a cool phase of the AMO should lead to a progressive delay in the mean birthdates in our study colony.

Coulson (1981) proposed that birthdates in grey seals are determined by regional SSTs which serve as a signal for the termination of the period of delayed implantation. We found a weak correlation between SST in the 3 months immediately after mating and the subsequent birthdate. However, SST was dropped from the model in favor of the population size, AMO and NAO indices, suggesting limited support for Coulson's hypothesis. It remains possible that grey seals have a direct physiological response to SST; however, as noted by Boyd (1984), there is little evidence that temperature alone can elicit the type of responses implied in Coulson's hypothesis. More likely Coulson's hypothesis serves to highlight the importance of the seasonality of the local environment on reproduction (Boyd, 1991).

The population size at our study colony was strongly correlated with the AMO index making it difficult to resolve their separate effects on birthdate. However, as the AMO appears to have been responsible for favorable environmental conditions throughout the course of our study, it seems likely that the increase in population size largely reflects favorable foraging conditions for females, leading to earlier birthdates. This kind of positive response to climate forcing has been reported in common eiders (*Somateria mollissima*) in Iceland where advancing laying date and increasing population size have been observed (D’Alba, Monaghan, & Nager, 2010).

### 4.2 Response of individuals to climate forcing

Within each breeding season, the distribution of grey seal births is quite synchronous but individual births within a season can occur over a period of as much as 45 days. Our results demonstrate a high level of repeatability (ICC = 0.66) within females such that most of the observed between‐seal variance is accounted for by maternal identity (i.e., traits of individual females). The finding that dates of parturition for individual females cover a significantly narrower range than for the population seems to be widespread (pinnipeds—Ellis, Bowen, Boness, & Iverson, 2000, Cordes & Thompson, 2013, Rotella et al., 2016, birds—Sydeman & Eddy, 1995, Thorley & Lord, 2015: ungulates—Plard, Bonenfant, Delorme, & Gaillard, 2012).

Although high repeatability in birthdates suggests a low level of plasticity for this trait in grey seals, as suggested in roe deer (Plard et al., 2012), we found evidence of significant individual plasticity in response to rapid climate forcing. Similarly, using a 47‐year study of the great tit (*Parus major*), Charmantier et al. (2008) showed that individual adjustment in behavior enabled the population to track a rapidly changing environment. Long‐term studies of individuals have revealed heterogeneity in the response to climate variability, termed IXE (individual–environment interaction, Nussey, Wilson, & Brommer, 2007), with certain individuals being more plastic in their phenology than others. The results from our long‐term study of individual grey seal females provide support for both rapid and heterogeneous response of individuals to climate change, with inexperienced females being most sensitive and experienced females being less sensitive but exhibiting more variable responses. Individual variation in the shape of the reaction norm of laying date also has been reported in birds (Nussey, Clutton‐Brock, Albon, et al., 2005; Nussey, Clutton‐brock, Elston, et al., 2005; Reed et al., 2009). Both the magnitude and speed with which the change in mean birthdates has occurred in grey seals suggest that phenotypic plasticity is most likely to account for the observed changes. As pedigree information is not available for this population, we cannot investigate whether there has been selection on birthdates. In red squirrels, parturition date was heritable and under phenotypic selection across the two decades of study; however, the early advance in birth dates reversed in the second decade. Selection did not act on the genetic contribution to variation in parturition date. Rather, as we expect is the case in our study, environmental variation and high food production in the first decade of the red squirrel study appears to have caused the shift in dates (Lane et al., 2018).

### 4.3 Consequences of changes in phenology

In many species, shifts in breeding phenology can distort the critical synchrony between young and their food supply (Visser, Both, & Lambrechts, 2004). Nevertheless, in many cases, it remains difficult to interpret changes in phenology with respect to fitness consequences (Visser & Both, 2005). The magnitude of the shift in our study means that grey seal pups will undertake their first foraging trip some 2 weeks earlier in 2017 than would have been the case three decades ago. The impact of this change on the fitness of offspring is difficult to determine as we know little about the initial diet of grey seals during this critical transition to nutritional independence. However, over the period of our study, juvenile survival from ages 0–4 years declined from 74% in the early 1990s to 33% in the early 2000s (den Heyer, Bowen, & McMillan, 2013). There is circumstantial evidence also from the at‐sea distribution of juveniles and adult females that adult females may displace juveniles from preferred foraging areas which could contribute to reduced juvenile survival (Breed, Bowen, & Leonard, 2013). Nevertheless, despite this reduced juvenile survival, the population has continued to increase. Therefore, it becomes problematic to disentangle the effects of shifting phenology on the availability of prey to young from the potential increased competition for food in a growing population.

Temporal changes in phenology resulting from climate change have often been associated with negative effects on reproductive performance or survival (Forcada & Hoffman, 2014; Parmesan, 2006). The advance of some 15 days in birthdates in our increasing population had no discernable effects on offspring body mass at weaning. In grey seals, survival to recruitment is a positive function of body mass at weaning (Hall et al., 2001, Bowen et al., 2015). The lack of change in weaning mass despite advancing phenology indicates that, to date, the changes in climate may not have resulted in changes in offspring fitness. Similarly, in red deer, despite advancing birthdates over a 40‐year period, there was no change in offspring birth mass or juvenile survival (Moyes et al., 2011; Stopher et al., 2014). This stasis in offspring traits in the face of changing phenology resulted from the counteracting effects of weather, population density, and maternal traits on birthdate, such that changes in climate did not generate changes in juvenile fitness (Stopher et al., 2014). As with grey seals in our study, in Common Eiders advancing breeding phenology has been associated with a growth in population size (D’Alba et al., 2010). The results from other taxa along with our findings indicate that the consequences of changes in phenology due to climate change will depend on how climate effects interact demographic and maternal traits. Shifts in breeding phenology need not have negative consequences.

## CONFLICT OF INTEREST

None declared.

## AUTHOR CONTRIBUTIONS

**William Don Bowen:** Conceptualization (lead); formal analysis (equal); funding acquisition (lead); investigation (equal); methodology (lead); writing–original draft (lead); writing–review and editing (equal). **Cornelia E. den Heyer:** Data curation (lead); formal analysis (lead); funding acquisition (supporting); investigation (equal); methodology (equal); writing–review and editing (equal). **Shelley Lang:** Investigation (equal); methodology (equal); writing–review and editing (equal). **Damian Lidgard:** Investigation (equal); methodology (equal); writing–review and editing (equal). **Sara J. Iverson:** Funding acquisition (supporting); investigation (equal); methodology (equal); writing–review and editing (equal).

## ETHICAL APPROVAL

All procedures used on study animals complied with applicable animal care guidelines of the Canadian Council on Animal Care and were approved by The Department of Fisheries and Oceans Animal Care Committee and the Dalhousie University Animal Care Committee.

## Supporting information

Supplementary Material

## Data Availability

Data used in our study are deposited with Dryad and can be accessed at https://doi.org/10.5061/dryad.j3tx95xbh.
