# Supplementary material for: Exploring causal components of plasticity in grey seal birthdates: Effects of intrinsic traits, demography, and climate
Source: Ecol Evol. 2020 Sep 28;10(20):11507–22. doi: 10.1002/ece3.6787 (PMC7593198; doi:10.1002/ece3.6787)

Supplemental material

Supplemental 1. The number of pups with known birthdates each year by cohort of adult females.

| **Year** | **1969** | **1970** | **1973** | **1974** | **1985** | **1986** | **1987** | **1989** | **1998** | **1999** | **2000** | **2001** | **2002** | **Sum** |
| --- | --- | --- | --- | --- | --- | --- | --- | --- | --- | --- | --- | --- | --- | --- |
| 1991 | 0 | 0 | 0 | 0 | 7 | 12 | 11 | 0 | 0 | 0 | 0 | 0 | 0 | 30 |
| 1992 | 0 | 0 | 6 | 1 | 16 | 18 | 18 | 0 | 0 | 0 | 0 | 0 | 0 | 59 |
| 1993 | 0 | 0 | 15 | 7 | 13 | 2 | 3 | 13 | 0 | 0 | 0 | 0 | 0 | 53 |
| 1994 | 0 | 0 | 4 | 0 | 15 | 6 | 2 | 2 | 0 | 0 | 0 | 0 | 0 | 29 |
| 1995 | 1 | 5 | 6 | 4 | 14 | 10 | 4 | 3 | 0 | 0 | 0 | 0 | 0 | 47 |
| 1996 | 3 | 7 | 23 | 6 | 36 | 34 | 13 | 12 | 0 | 0 | 0 | 0 | 0 | 134 |
| 1997 | 1 | 7 | 17 | 6 | 33 | 25 | 12 | 4 | 0 | 0 | 0 | 0 | 0 | 105 |
| 1998 | 2 | 9 | 20 | 17 | 33 | 30 | 13 | 12 | 0 | 0 | 0 | 0 | 0 | 136 |
| 1999 | 0 | 10 | 11 | 10 | 15 | 22 | 9 | 6 | 0 | 0 | 0 | 0 | 0 | 83 |
| 2000 | 1 | 10 | 22 | 17 | 33 | 43 | 23 | 18 | 0 | 0 | 0 | 0 | 0 | 167 |
| 2001 | 2 | 10 | 24 | 13 | 39 | 39 | 17 | 17 | 0 | 0 | 0 | 0 | 0 | 161 |
| 2002 | 2 | 10 | 22 | 11 | 40 | 37 | 22 | 15 | 0 | 0 | 0 | 0 | 0 | 159 |
| 2003 | 0 | 8 | 15 | 10 | 33 | 33 | 20 | 18 | 11 | 0 | 0 | 0 | 0 | 148 |
| 2004 | 1 | 6 | 15 | 12 | 31 | 35 | 21 | 15 | 13 | 21 | 0 | 0 | 0 | 170 |
| 2005 | 1 | 3 | 8 | 6 | 16 | 16 | 8 | 6 | 10 | 14 | 10 | 2 | 0 | 100 |
| 2006 | 1 | 3 | 5 | 7 | 23 | 25 | 10 | 8 | 12 | 42 | 20 | 18 | 4 | 178 |
| 2007 | 1 | 0 | 7 | 5 | 19 | 14 | 9 | 7 | 16 | 30 | 17 | 18 | 9 | 152 |
| 2008 | 0 | 1 | 7 | 5 | 11 | 15 | 6 | 6 | 11 | 20 | 18 | 22 | 17 | 139 |
| 2009 | 0 | 1 | 3 | 4 | 14 | 12 | 8 | 8 | 10 | 23 | 10 | 10 | 15 | 118 |
| 2010 | 0 | 0 | 1 | 3 | 15 | 16 | 9 | 5 | 14 | 19 | 13 | 10 | 10 | 115 |
| 2011 | 0 | 0 | 1 | 0 | 7 | 15 | 5 | 10 | 3 | 10 | 9 | 11 | 7 | 78 |
| 2012 | 0 | 0 | 0 | 2 | 11 | 13 | 5 | 5 | 15 | 17 | 16 | 13 | 13 | 110 |
| 2013 | 0 | 0 | 2 | 1 | 8 | 11 | 7 | 4 | 5 | 10 | 7 | 13 | 7 | 75 |
| 2014 | 0 | 0 | 1 | 1 | 5 | 6 | 2 | 4 | 5 | 18 | 8 | 14 | 8 | 72 |
| 2015 | 0 | 0 | 0 | 0 | 2 | 3 | 1 | 2 | 3 | 2 | 3 | 5 | 3 | 24 |
| 2016 | 0 | 0 | 0 | 0 | 7 | 6 | 5 | 4 | 9 | 15 | 14 | 11 | 11 | 82 |
| 2017 | 0 | 0 | 0 | 0 | 1 | 3 | 2 | 2 | 3 | 7 | 12 | 7 | 7 | 44 |

Supplemental 2. Histogram of the number of pups with known birthdates for each mother.


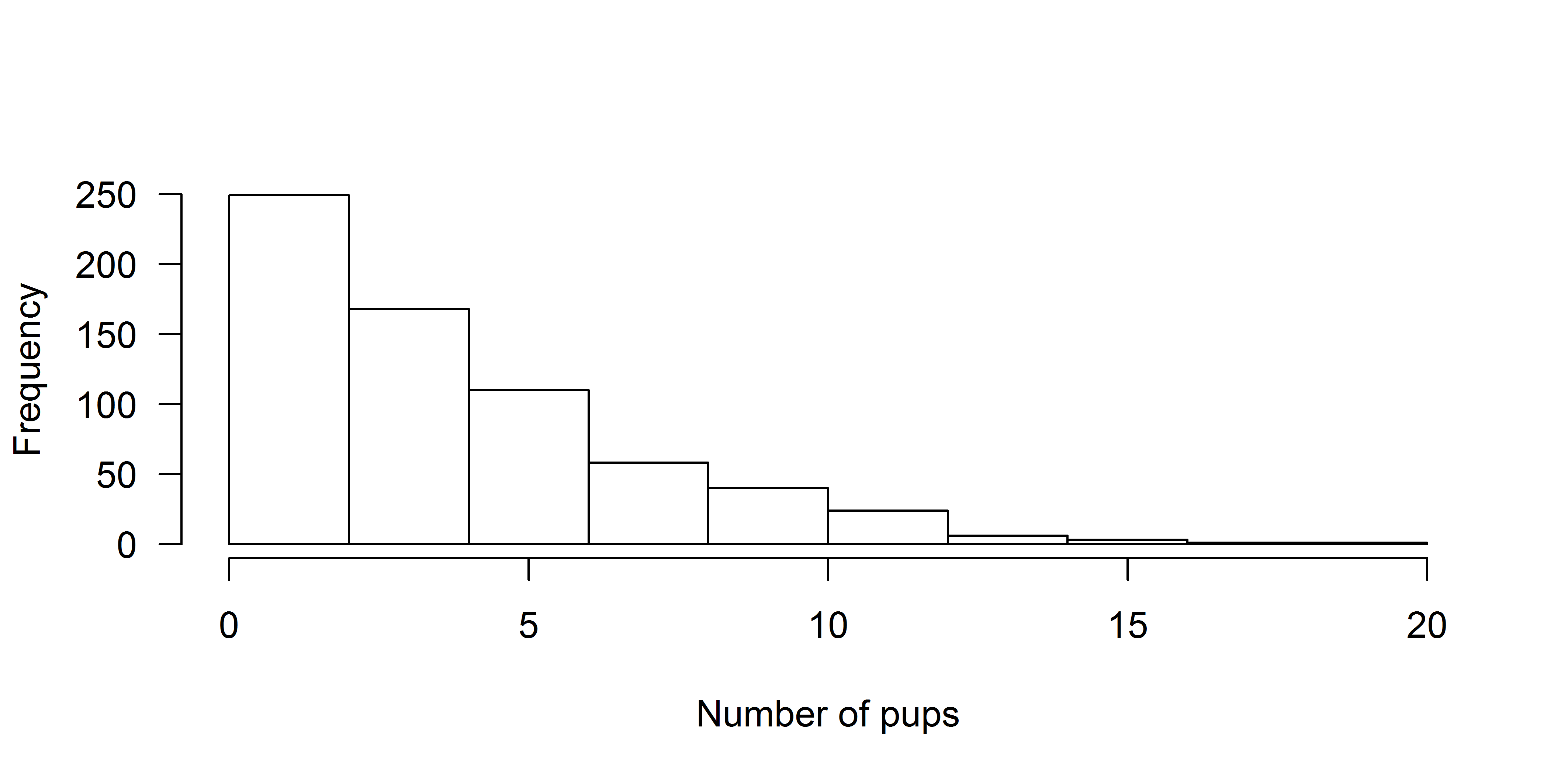


Supplemental 3. Parameter estimates the mixed-effect model of pup birthdate, including mother's number of previous pups (Parity), mother's pupping success in previous year (Success), mother's age (Age) which was normalized and included as quadratic, the sex of the pup (female), and year as a factor.

|  | **Estimate** | **Std. Error** | **t value** |
| --- | --- | --- | --- |
| Intercept | 43.5078 | 0.745899 | 58.32932 |
| Parity (2) | -3.01077 | 0.387283 | -7.77409 |
| Parity (3+) | -4.3617 | 0.392203 | -11.121 |
| Pup sex (female) | 0.720564 | 0.137315 | 5.247534 |
| Previous Success (true) | -0.63339 | 0.165866 | -3.81872 |
| Age | 0.053343 | 0.064986 | 0.820833 |
| I(Age^2) | 0.006908 | 0.00152 | 4.543613 |
| Year1992 | -4.68435 | 0.802231 | -5.83915 |
| Year1993 | -3.02654 | 0.841921 | -3.59481 |
| Year1994 | -1.47867 | 0.942594 | -1.56873 |
| Year1995 | -2.70674 | 0.901593 | -3.00217 |
| Year1996 | -5.29711 | 0.778145 | -6.80736 |
| Year1997 | -6.45092 | 0.798323 | -8.08059 |
| Year1998 | -7.77981 | 0.788092 | -9.87171 |
| Year1999 | -8.62734 | 0.823043 | -10.4822 |
| Year2000 | -11.9255 | 0.783961 | -15.2119 |
| Year2001 | -11.1107 | 0.792727 | -14.0158 |
| Year2002 | -10.1136 | 0.801081 | -12.6249 |
| Year2003 | -12.3 | 0.806345 | -15.254 |
| Year2004 | -11.0621 | 0.804023 | -13.7584 |
| Year2005 | -11.7659 | 0.837987 | -14.0407 |
| Year2006 | -13.0927 | 0.81019 | -16.1601 |
| Year2007 | -13.7455 | 0.833921 | -16.483 |
| Year2008 | -13.4658 | 0.852039 | -15.8042 |
| Year2009 | -13.6236 | 0.884737 | -15.3985 |
| Year2010 | -14.8029 | 0.898269 | -16.4794 |
| Year2011 | -15.6931 | 0.955232 | -16.4286 |
| Year2012 | -14.6591 | 0.928535 | -15.7874 |
| Year2013 | -13.9194 | 0.970044 | -14.3492 |
| Year2014 | -16.0938 | 0.993927 | -16.1921 |
| Year2015 | -16.5026 | 1.160835 | -14.2162 |
| Year2016 | -14.5037 | 1.015599 | -14.281 |
| Year2017 | -16.424 | 1.093711 | -15.0168 |

Supplemental 4. Post-hoc analysis to identify parity plus bin size. The model of pup birthdate includes mother's parity (*β_parity_*), mother's pupping success in previous year (*β_suc_*), mother's age (*β_age_* + *β_age2_*) which was normalized and included as quadratic, pup sex (*β_sex_*) and year as a factor (1991 to 2017). The 4 models included parity as factor with 2+, 3+, 4+ and 5+ bins.

| **Model names** | **K** | **AICc** | **Delta AICc** | **Model**  **Likelihood** | **AICc Wt** | **LL** | **Cum. Wt** |
| --- | --- | --- | --- | --- | --- | --- | --- |
| Parity (1,2, & 3+) | 36 | 15519.75 | 0 | 1 | 0.435175 | -7724.4 | 0.435175 |
| Parity (1,2,3 &4+) | 37 | 15519.85 | 0.094798 | 0.953707 | 0.415029 | -7723.42 | 0.850204 |
| Parity (1,2,3,4 &5+) | 38 | 15521.9 | 2.149977 | 0.341302 | 0.148526 | -7723.42 | 0.99873 |
| Parity (1 & 2+) | 35 | 15531.42 | 11.67331 | 0.002919 | 0.00127 | -7731.27 | 1 |

Supplemental 5. Plot the simulated A) fixed and B) random effects from the mixed-effect model of pup birthdate relative to December 1, including mother's parity (Parity), mother's pupping success in previous year (Success), mother's age (Age) which was normalized and included as quadratic, the sex of the pup (Pup Sex), AMO, 3 year mean AMO, NAO and 3 year mean NAO and individual seal (momid) as a random effect (*ϒ*_j_) .


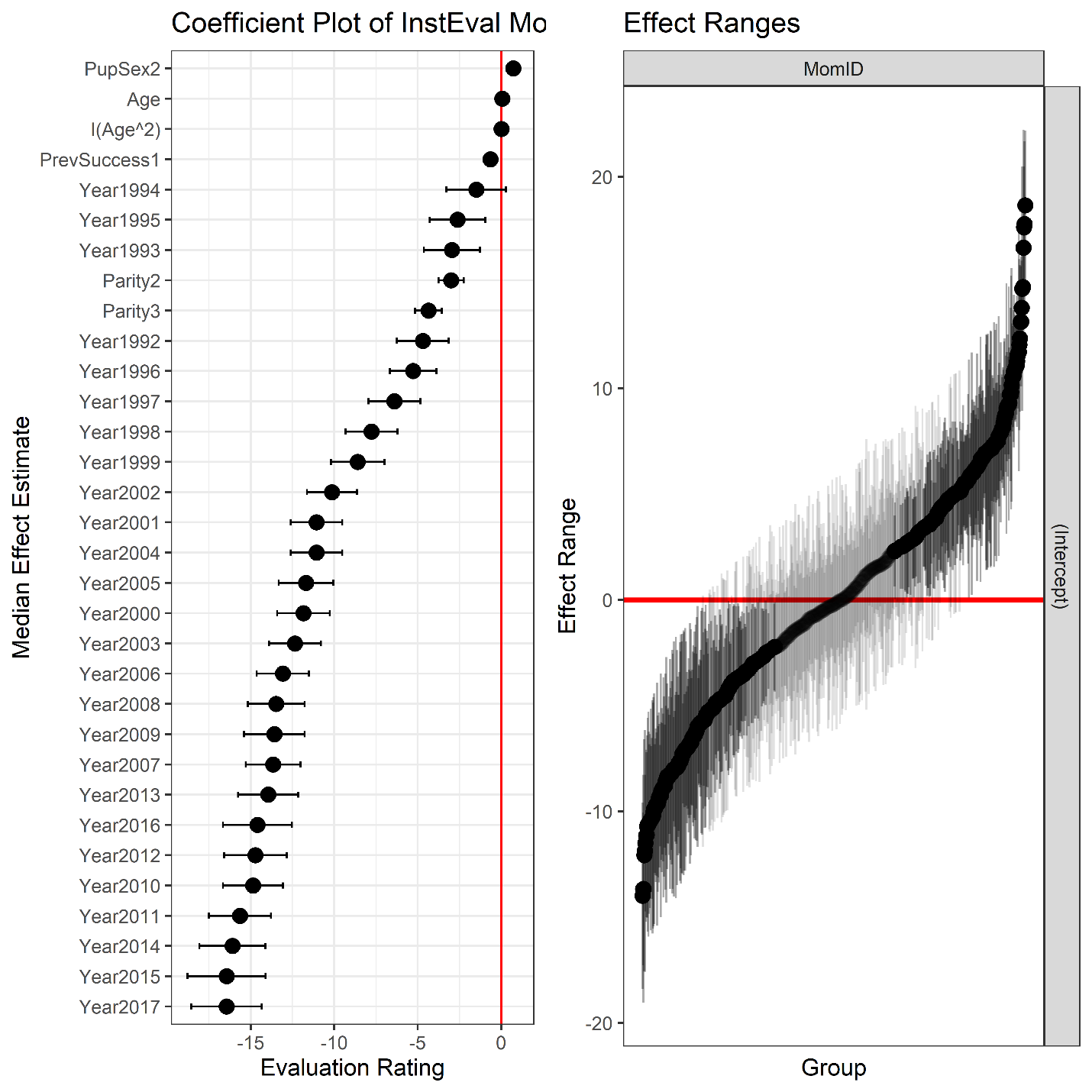


Supplemental 6. Post-hoc analysis to identify parity plus bin size for mixed-effect model of pup weaning mass, including mother's parity (Parity), mother's pupping success in previous year (Previous Success), mother's age (Age) which was normalized and included as quadratic, the sex of the pup and the birthdate anomaly for that pup. Parity is included as factor with 2+, 3+, 4+ and 5+ bins.

| **Model names** | **K** | **AICc** | **Delta_AICc** | **Model Likelihood** | **AICc Wt** | **LL** | **Cum .Wt** |
| --- | --- | --- | --- | --- | --- | --- | --- |
| Parity (1,2, & 3+) | 10 | 14012.14 | 0 | 1 | 0.533837 | -6996 | 0.533837 |
| Parity (1,2,3 &4+) | 11 | 14013.17 | 1.045539 | 0.592876 | 0.3165 | -6995.51 | 0.850337 |
| Parity (1,2,3,4 &5+) | 12 | 14014.66 | 2.543411 | 0.280353 | 0.149663 | -6995.25 | 1 |
| Parity (1 & 2+) | 9 | 14057.54 | 45.30489 | 1.45E-10 | 7.75E-11 | -7019.66 | 1 |

Supplemental 7. Plot the simulated random effects from the for mixed-effect model of pup weaning mass, including mother's parity (Parity with 3 bins), mother's pupping success in previous year (Previous Success), mother's age (Age) which was normalized and included as quadratic, the sex of the pup and the birthdate anomaly for that pup.


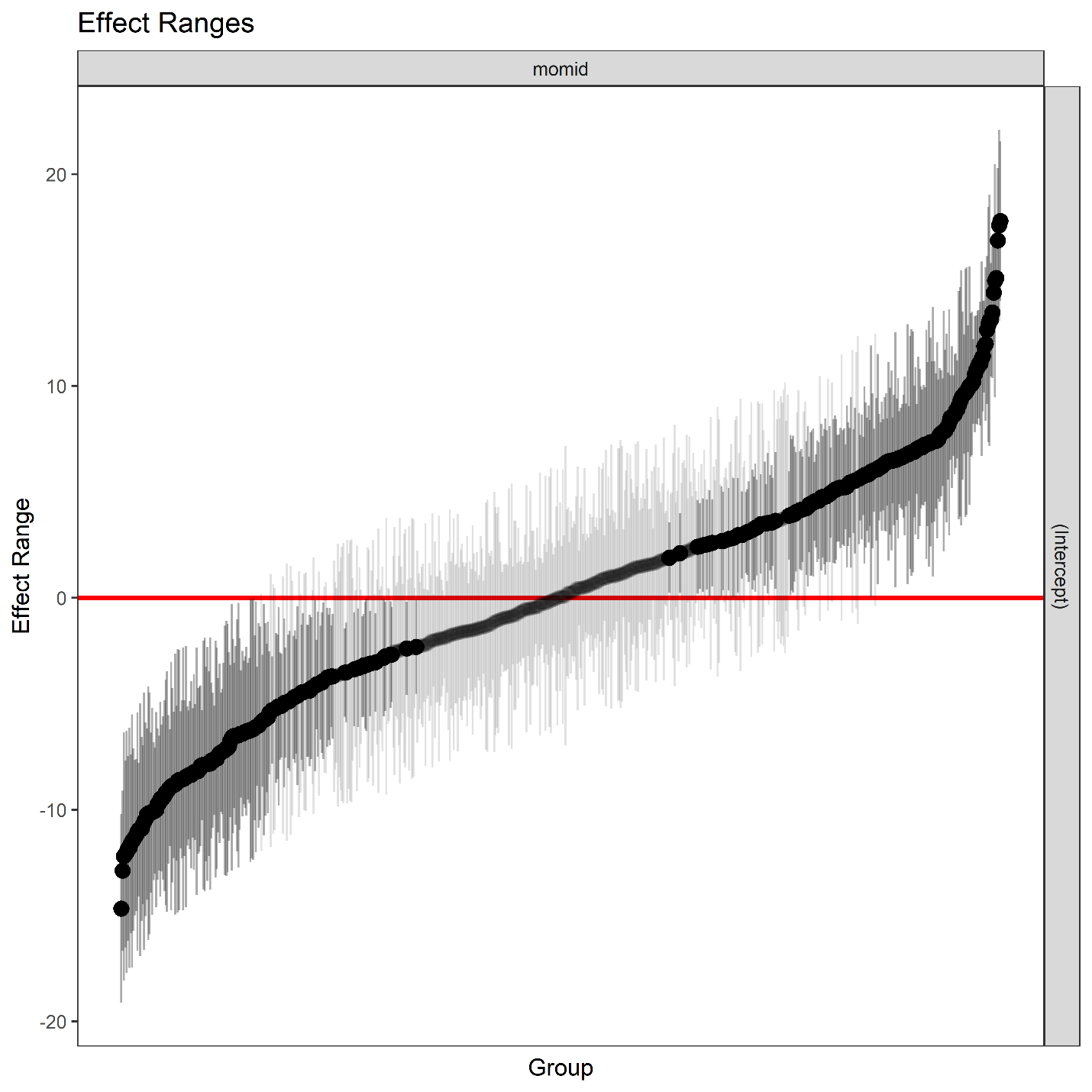

Supplement: Supplementary file 1 — Supplementary Material [file ECE3-10-11507-s001.docx]
